# Hypoxia-related mechanisms inducing acute mountain sickness and migraine

**DOI:** 10.3389/fphys.2022.994469

**Published:** 2022-09-06

**Authors:** Florian Frank, Katharina Kaltseis, Vera Filippi, Gregor Broessner

**Affiliations:** Innsbruck Medical University, Department of Neurology, Innsbruck, Austria

**Keywords:** hypoxia, AMS, HAH, migraine, human migraine model, CGRP

## Abstract

Experimental models of human diseases are vital for pathophysiological and therapeutic research. To investigate the initiation, maintenance, pathophysiology and even termination of a migraine/headache attack these models are urgently needed. Results from different studies promote the profound involvement of hypoxia in migraine and other primary/secondary headaches. The possible mechanisms that drive the induction of headaches through hypoxia are still unknown, but several modes of action, such as increased blood flow, dilation of cerebral arteries, the release of nitroglycerin, calcitonin gene-related peptide and adenosine or increased oxygen extraction are discussed intensively. In studies exposing healthy volunteers and people with a history of migraine to controlled normobaric hypoxia, our research group could demonstrate normobaric hypoxia to be an effective trigger of migraine headaches. Furthermore, a longitudinal measurement of calcitonin gene-related peptide (CGRP), during a hypoxic challenge in migraine patients, revealed increasing CGRP levels with prolonged hypoxic challenge. Since GRP has been linked to migraine and other headache disorders, hypoxia could be regarded as initiator for headaches on a neurotransmitter basis. Furthermore, it has been known for more than 2 decades from studies *in vitro* and *in vivo* that hypoxia can induce cortical spreading depression, a phenomenon believed to represent aura. Considering the increased prevalence of migraine in altitude populations and the solid pathophysiological changes on cellular and neurotransmitter level–the role of hypoxia should be investigated in greater detail by the headache community.

## 1 Introduction

### 1.1 Hypoxia related mechanisms inducing acute mountain sickness and migraine

Hypoxia is a state in which oxygen is not available in adequate amounts at the tissue level to maintain physiological homeostasis. It can either be the result of insufficient oxygen delivery to the target tissue, as in ischemic stroke and lead to focal deficits, or due to low oxygen saturation in the blood per se as a consequence of reduced oxygen exposure as in higher altitudes. It is very well established that hypoxia can cause secondary headache such as high-altitude headache (HAH), headache in combination with acute mountain sickness (AMS) or even headache attributed to airplane flights (Britze et al., 2016; Konrad et al., 2022). Headache that occurs with an increase in altitude is the cardinal symptom of AMS and is usually accompanied by vegetative symptoms such as anorexia, nausea, dizziness, malaise, sleep disturbance, or a combination of all these symptoms (Bärtsch et al., 2004; Bärtsch and Swenson, 2013). AMS occurs in 50–80% of unacclimatized persons who ascend to 2500 m or higher making AMS also a relevant disease entity in moderate altitude regions such as the alps (Bärtsch and Swenson, 2013). If AMS is neither diagnosed nor treated, high-altitude cerebral edema (HACE) characterized by truncal ataxia, decreased consciousness, and usually mild fever, may become a life-threatening condition with rapidly evolving coma (Bärtsch and Swenson, 2013).

Most interestingly the headache features of AMS include many migraine features and several groups including our own have shown that a migraine attack can be triggered by hypoxia even in migraine naive persons (Burtscher et al., 2010, 2014; Bärtsch and Swenson, 2013). Furthermore, it could be shown that Sumatriptan is efficacious in treating HAH, migraine and preventing AMS (Bärtsch et al., 1994; Jafarian et al., 2007) This has led to the assumption that hypoxia may play an important role in the development of primary headaches as well. Indeed, results from studies could show an involvement of hypoxia not only in migraine but also in cluster headache (Cohen et al., 2009). However, the possible mechanisms that drive the induction of primary/secondary headaches through hypoxia are still unknown–but several modes of action are discussed intensively: 1) increased blood flow, 2) dilation of cerebral arteries, 3) release of nitric oxide (NO), CGRP and adenosine 4), increased oxygen extraction from blood, 5) possible disruption of the blood brain barrier (BBB), 6) release of hypoxia-induced gene transcription factors (HIF) promoting downstream adenosine triphosphate (ATP) synthesis and angiogenesis (Arngrim et al., 2016; Britze et al., 2016). Through evolution, the brain has developed a variety of highly adaptive mechanisms for hypoxia, both in the short and long term. Partial pressures of Oxygen and carbon dioxide, temperature and potential of hydrogen (pH) value are constantly sensed in certain parts of the vasculature and consequently adjusted to avoid disbalance (Bärtsch and Swenson, 2013; Britze et al., 2016). As soon as the peripheral chemo sensors detect an arterial oxygen partial pressure below 60 mmHg a whole wave of counterregulatory mechanisms are initiated involving central (hypothalamus, nucleus of the solitary tract (NTS), paraventricular nucleus (PVN), ventral/dorsal respiratory group) and peripheral (glossopharyngeal nerve, vagal afferents) pathways (Kc and Dick, 2010; Britze et al., 2016). This regulatory circuit enables adaption of ventilatory rate, oxygen extraction rate, cerebral blood flow and release of neuropeptides as mentioned above. If all those measures fail or are at least insufficient the brain can produce energy anaerobically via glycolysis with the generation of lactate. If this accumulation of lactate may be the promotor for primary or secondary headaches is currently debated (Harris et al., 2013).

Although the underlying mechanisms are not entirely clear up to date, the effect of hypoxia is so profound that it can be observed even on the epidemiological level. First, high-altitude regions are associated with a higher prevalence rate of migraine (Arregui et al., 1991; Linde et al., 2017). This rise in prevalence even seems to be correlated with the extent of hypoxia, as another lower high-altitude region with a comparable socioeconomic population showed a lower prevalence (Jaillard et al., 1997). Second, AMS and HAH are found in up to 80% of mountaineers and hikers traveling above 2,500 m above sea level. Astoundingly, the characteristics of migraine and HAH are partially overlapping and migraine appears to be an independent risk factor for the development of HAH as shown by two prospective trials including 1,326 and 506 participants respectively (Burtscher et al., 2010; Canouï-Poitrine et al., 2014). These findings have led to the investigation of sumatriptan as a possible common treatment of migraine and HAH. However, results were discrepant among studies, possibly due to the clinical overlap in both entities. (Bärtsch et al., 1994; Utiger et al., 2022; Burtscher et al., 1995). The similarities between HAH and migraine apparently are not only present within the diagnostic criteria proposed by the International Classification of Headache Disorders, 3rd edition (ICHD-III) (Headache Classification Committee of the International Headache Society IHS, 2018). In a study exposing healthy volunteers to normobaric hypoxia our research group could demonstrate that migraine-like features are present during the hypoxic challenge in a population even without any history of migraines (Broessner et al., 2015). Rather elusive to clinical research, migraine aura has shown pathophysiological links to hypoxia, as animal studies demonstrated tissue hypoxia to be causes and associated with cortical spreading depression (Somjen et al., 1992; Takano et al., 2007).

Importantly the influence is not unidirectional but rather bidirectional as migraine per se is a risk factor for HAH and AMS. In a prospective study investigating predictors of HAH and AMS in mountaineers at high altitude, our group could show that a history of migraine was the most important factor, increasing the risk for HAH by 6-fold (Burtscher et al., 2010).

However, the situation is complicated by the fact that many similarities between the clinical manifestations of migraine, HAH and AMS make it difficult to determine whether these numbers reflect an increased susceptibility to secondary headaches among migraineurs, or genuine migraine attacks that are in fact provoked by the high-altitude environment (Britze et al., 2016).

## 2 Hypoxia as a human model for migraine induction

The pivotal rationale for performing research in the clinical medical field is to illuminate (patho)-mechanisms underlying a disease entity. In particular, targeted treatments are entirely reliant on formulating pathways that elucidate the molecular, physiological, and clinical background. To obtain this knowledge disease models proved to be a valuable tool, *in vitro* as *in vivo*.

Migraine, being an affliction that is invariably diagnosed by patient report, has recently received targeted therapeutic measures with the introduction of monoclonal antibodies against calcitonin gene-related peptide (CGRP) or its receptor. The development of this milestone in migraine therapy was carried in large part by experimental models. *In-vitro* and animal models provide invaluable information on molecular and cellular interactions involved in a migraine attack. This has been clearly shown by animal studies investigating the trigeminovascular complex, cortical spreading depression (CSD) and in general mechanisms of nociception (Noseda and Burstein., 2013). However, to further elucidate the clinical presentation of a spontaneous migraine attack, aura or prodromal phase and–even more–the efficacy of pharmacological agents, human models are required. A major reason why animal models are unsuited for this purpose is the observation, that migraine patient brains show differences in morphology as well as functionality (DaSilva et al., 2007; Schulte and May 2016).

Quite a number of neurological disorders are in fact episodic, which requires a differentiated approach to modelling a disease. Chronic neurological disorders can be explored throughout a longer course of the disease, rendering the exact timing of the investigation secondary. Due to the ictal nature of migraine, research in this field often is more time sensitive and requires ‘tipping the scales’ or providing the energy of activation to induce a migraine state” (Demartini et al., 2019). On this account, a human migraine model to induce a migraine attack reliably and safely is favoured over basic observations. Considering the diagnostic criteria for migraines, as laid out by ICHD-III (Headache Classification Committee of the International Headache Society IHS, 2018), an experimentally induced migraine attack will not fulfill the definition of a natural migraine. However, by achieving a close approximation to the symptoms reported during usual migraines, human models can still provide a potent surrogate.

A somewhat natural migraine model is inherent in patients suffering from familial hemiplegic migraines (FHM). This group of disorders is characterized by hemiplegic, basilary or cerebellar aura and is linked to monogenic mutations. The pathophysiology of FHM has been studied with interest to explore an association between pathology and phenomenology. However, triggering agents that are used for “regular” migraine attacks have not been as successful in FHM (Hansen et al., 2008), indicating that FHM might possess different pathophysiological mechanisms, that ultimately lead to a brain state of higher excitability.

Perhaps the earliest human migraine model was introduced in the past century with nitroglycerine (NTG). Italian chemist Ascanio Sobrero synthesized NTG by pursuing his mentors research on the explosive nitrocellulose. After digesting minimal amounts of the compound, he experienced “a violent headache for several hours” (Hughes and Dake., 1888). In subsequent years the beneficial effects of NTG as antihypertensive drug and relieve of angina pectoris displaced the interest of its use as a headache trigger. Almost a hundred years later, with the comprehension of NTGs mechanisms as inducers of NO release, it became relevant to headache research again. This interest was primarily inspired due to the prevalent vascular theory of migraine at that time. Even after the thriving dismissal of this theory and its replacement with the neurogenic concept, NTG as migraine model possesses significance. It is discussed, that NTG may contribute to mechanisms further downstream, such as sensitization of nociceptive networks and receptors as well as activation of pain-promoting pathways (Moskowitz, 1993; Reuter et al., 2001; Benemei et al., 2013). A pivotal nexus in this scenario is the activation of channels of the transient receptor potential (TRP) family. The ankyrin and vanilloid subtypes (TRPA, TRPV) are targeted by various stimuli among them NO, O3, H+, and other reactive oxygen species. Upon stimulation, voltage-gated cation channels are opened causing calcium inflow and subsequently the release of neuropeptides such as Calcitonin Gene-Related Peptide (CGRP) and Substance P. These peptides are thought to play a major role in the sensitization of nociceptive neurons (Goadsby et al., 2017).

Human headache studies demonstrated an immediate and rather short-lasting headache after NTG administration in healthy volunteers (Iversen, 1995). When administered to migraine patients most (<60%) (Sances et al., 2004) still experience an unspecific fast onset headache caused by NTG or no pain at all. However, after two to 6 hours after NTG administration migraine patients (around 20–80%) (Sicuteri et al., 1987; Thomsen and Olesen., 1997; Schoonman et al., 2006) develop a delayed headache resembling their usual migraine attack, independent of their baseline attack frequency. In contrast, healthy volunteers do not seem to display the delayed headache response at all. The delayed onset of migraine headache might be explained by the prerequisite of sensitized neuronal pathways as pointed out in the previous paragraph. Interestingly, patients suffering from migraine with aura develop NTG triggered migraine attacks less likely (40–60%) (Christiansen et al., 1999; Sances et al., 2004) compared to patients with migraine without aura. Furthermore, a review on experimental migraine provocation methods revealed that migraine aura can only be triggered in only four to 14% utilizing NTG (Sances et al., 2004). The NTG provocation test still is a powerful tool in migraine research, however, due to the low success rates in triggering migraines in patients with migraine aura, direct vascular activation leading to severe hypotonia, and the low rates of elicited migraine auras offers room for improvement.

As mentioned above, a common pathway of NTG in migraine pathophysiology might be sensitization of central and peripheral neuronal structures by reactive oxygen species and uncoupling of nitric oxide synthases (NOS), resulting in a common terminal path with brain energy deficit as potential source of oxidative stress (Borkum, 2021). Hypoxia as possible pathogenic mechanism in migraine has been proposed in the early 90s by Amery postulating migraines might be a reaction to cerebral hypoxia (Amery, 1982; Amery, 1985). Amery even went as far as to provocatively stating: “NO HYPOXIA, NO MIGRAINE”. Also, several commonly known migraine triggers are considered as potential donors of reactive oxygen species, resulting in a hypoxic cellular environment (Borkum, 2016). Clinical studies have shown that hypoxia might also be of importance in the pathology of headaches occurring in high altitude. *In-vivo* human studies have already shown that hypoxia is capable of triggering migraine attacks. Schoonman et al. could elicit migraines in 42% of 16 participants by hypoxia, whereas NTG was only half as effective (21%) (Schoonman et al., 2006). In a similar fashion, by utilizing breathing masks with reduced FiO2, Arngrim et al. provoked migraine headache in 8 out of 15 subjects (53%). They used a population of patients with migraine with aura only and could also trigger migraine aura in 20% of their subjects, reinforcing the association of CSD and tissue hypoxia. To address the issue of hypoxia-induced migraine to be either of vascular or neural origin, Arngrim et al. studied vessel diameters during their experiment, concluding that hypoxia-induced migraines are of metabolic origin, as cerebral vasodilatation did not differ significantly between groups (Arngrim et al., 2016).

**FIGURE 1:**
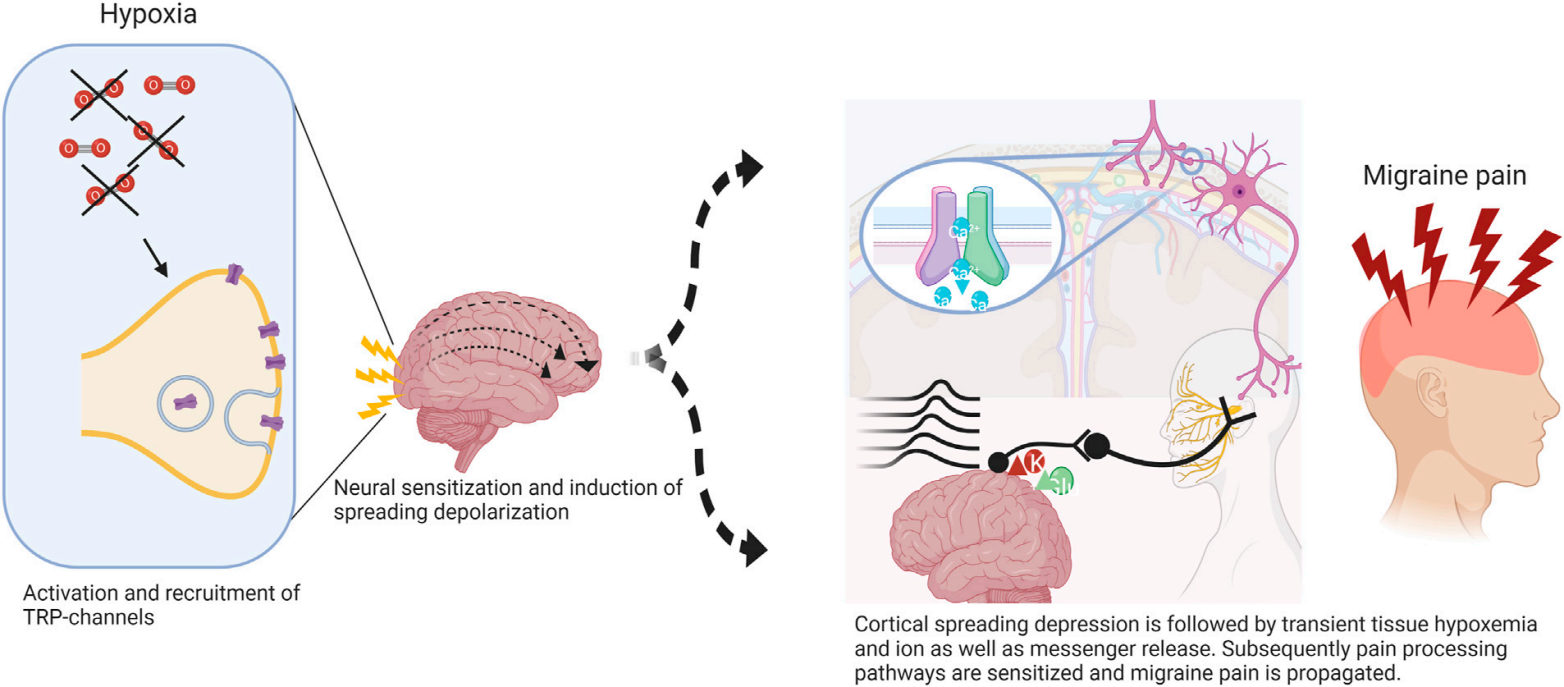
Potential mechanism of hypoxia-induced cortical spreading depression (CSD) in migraine. Normobaric hypoxia can cause activation of redox-sensitive transient receptor potential channels (TRP) with further insertion of transmembrane proteins involved in signal propagation. Trigeminal afferents become sensitized via meningeal nociceptors inducing higher activity in central projections promoting cortical excitation leading to CSD. The initial wave of depolarization followed by tissue hypoxemia results in further activation and sensitization of pain processing pathways and thus migraine pain. Adapted from (Frank et al., 2020).

Our group was able to demonstrate normobaric hypoxia to be an effective trigger of migraine headaches in 63.3% (*n* = 19) of our subjects. Comparably to Arngrim et al. we induced migraine aura in five (16.6%) subjects of a mixed population with and without migraine aura. To our own surprise, two of these subjects never experienced an aura before in their life, possibly indicating that any (migraine) brain is capable of developing aura once an individual threshold is surpassed, and neural rescue mechanisms are overwhelmed (Figure 1).

## 3 Course of migraine related biomarkers under hypoxia

Since decades, extensive research has been conducted investigating the complex and miscellaneous pathophysiology of migraine. Various pathways have been promoted and discarded over the past years, including the vascular theory–vasodilatation causing the pain in migraine headache – (Ray and Wolff., 1940) - and the hypothesis of neurogenic inflammation–bringing to the fore neuropeptides as mediators in migraine pathophysiology (Edvinsson et al., 2019). Thereof, especially agents with high vasoactive potential as CGRP and pituitary adenylate cyclase-activating polypeptide (PACAP) have become the focus in migraine research (Ashina et al., 2017; Ashina et al., 2021a).

### 3.1 Calcitonin gene-related peptide

CGRP has probably become the most important blood biomarker in migraine research since its discovery in the early 1980s (Rosenfeld et al., 1983). It is significance in migraine pathophysiology can be attributed to the following: 1) Intravenous application of CGRP can trigger migraine attacks, 2) studies investigated the ictal and/or interictal CGRP levels in saliva, tear fluid, cerebrospinal fluid and peripheral blood showed increased levels during a migraine attack (Goadsby et al., 1988; Van Dongen et al., 2017; Alpuente et al., 2022) and 3) new agents targeting CGRP or its receptor are effective in the prevention of migraine (Edvinsson, 2021).

CGRP, a 37 amino-acid neuropeptide, features a potent vasodilator activity and is expressed in two isoforms: *a*-CGRP and β-CGRP, with the *a*-isoform playing an important role in pain processing in the peripheral and central nervous system. It is released from trigeminal afferent fibers (Messlinger, 2018). As the synthesis of CGRP seems to be enhanced as part of an inflammatory response, higher levels during hypoxia induced oxidative stress could be suspected (Edvinsson et al., 2019). However, only few studies assessed peripheral CGRP levels during hypoxia–with conflicting results, probably due to heterogeneous protocols and inconsistent sample processing (Messlinger., 2018). In rodent studies, blood CGRP levels were decreased during hypoxia. (Keith and Ekman., 1992; Keith et al., 2000). A previous in-human study did not find altered peripheral blood CGRP levels in patients naïve to headache and migraine after 9 h of exposure to normobaric hypoxia (12.9% O2) (Bailey et al., 2009). Similarly, Hasbak et al. found no difference in CGRP levels at sea level compared to measurements at a high altitude of 4559 m (Hasbak et al., 2002). These studies have been carried out years before and the explanation of the results may be that CGRP production, release, degradation, and sampling procedures are far from trivial (Messlinger et al., 2018). First, only a minor fraction of CGRP appears in blood samples taken from cubital veins, especially if these are far away from the sites of CGRP release (i.e. trigeminal ganglion). Second, because CGRP is degraded immediately upon release by peptidases in the blood plasma with a half-life of about 7–10 min, only a fraction of the original concentration will appear at the site of blood sampling like the cubital veins (Kraenzlin et al., 1985). Third, the handling of enzyme-linked immunosorbent assays (ELISAs) is highly variable and has not been described precisely enough in most papers, so that it is hardly possible for other groups to repeat the respective measurements. Therefore, it is not astonishing that inconsistent data about the CGRP levels in human blood has been published, even in investigating the same disorders as migraine. To overcome this bias, we encourage the scientific community to use a standardized and evaluated CGRP sampling approach fulfilling international requirements which has been published in detail by Messlinger et al. (Messlinger et al., 2018).

Using this standardized ELISA based approach, in a longitudinal measurement of CGRP during a normobaric hypoxic challenge in migraine patients, we found elevated CGRP levels with increasing duration of the hypoxic challenge (Frank et al., 2022 in press).

### 3.2 Pituitary adenylate cyclase-activating polypeptide

PACAP is considered a significant neurotransmitter/neuromodulator in migraine pathophysiology and subsists in two forms, PACAP-27 and PACAP-38, the latter being the predominant form in neuronal tissue (Waschek et al., 2018).

As CGRP, PACAP is a potent local and systemic vasodilator, and its intravenous application can trigger delayed migraine-like attacks (Schytz et al., 2009). The blockade of PACAP in migraine patients has been investigated in a Phase II trial however with only negative results so far (Ashina et al., 2021b).

Regarding the ictal or interictal course of PACAP-38, two studies found elevated PACAP levels during a migraine attack (Tuka et al., 2013; Zagami et al., 2014). In contrast, two studies did not find increased interictal levels (Tuka et al., 2013; Cernuda-Morollón et al., 2016). Currently, there are no studies on the course of PACAP-38 during (controlled) hypoxia in migraine patients published.

## 4 Conclusion

Normobaric hypoxia, in addition to the NTG-infusion model, is a solid method of inducing a migraine attack in individuals with and without a history of migraine. Although an experimentally induced migraine attack does not fulfil the ICHD-3 criteria of a migraine attack technically speaking, the symptomatology described by study participants exposed to normobaric hypoxia, the reproducibility, and the elevation of one of the most important migraine biomarkers, CGRP, still suggest controlled normobaric hypoxia as a reliable and promising human migraine model. In studies exposing individuals with a history of migraine to controlled normobaric hypoxia, our research group could demonstrate an unimodal migraine-like headache and aura response (Frank et al., 2020). The features of the headache induced by normobaric hypoxia share many similarities with the migraine definition. The participants in this prospective study have been asked during the exposure if the induced headache reflects their typical migraine attack - with most of them confirmed. The normobaric hypoxia as a human migraine model has intriguing advantages towards the NTG model comprising a non-pharmacological intervention with little side effects, higher attack induction rates, no direct vascular interaction and increased aura induction. However, we are fully aware that larger well-controlled studies must be conducted to investigate if normobaric hypoxia is superior to the NTG approach as human migraine model. The underlying pathomechanisms of hypoxia-induced migraine/headache attacks are yet not entirely clear but based upon solid pathophysiological evidence including prominent neurotransmitters such as CGRP. We propose that the involvement of hypoxia in the pathophysiology of headache/migraine attacks should become a research focus in the headache community.

## References

[B1] Alpuente A. Gallardo V. J. Asskour L. Caronna E. Torres-Ferrus M. Pozo-Rosich P. (2022). Salivary CGRP can monitor the different migraine phases: CGRP (in)dependent attacks. Cephalalgia. 42 (3), 186–196. 10.1177/03331024211040467 34601944

[B2] Amery W. K. (1982). Brain hypoxia: The turning-point in the Genesis of the migraine attack? Cephalalgia 2 (2), 83–109. 10.1046/j.1468-2982.1982.0202083.x 6751554

[B3] Amery W. K. (1985). Migraine and cerebral hypoxia: A hypothesis with pharmacotherapeutic implications. Cephalalgia. 5, 131–133. 10.1177/03331024850050S224 4016926

[B4] Arngrim N. Schytz H. W. Britze J. Amin F. M. Vestergaard M. B. Hougaard A. (2016). Migraine induced by hypoxia: An MRI spectroscopy and angiography study. Brain 139, 723–737. 10.1093/brain/awv359 26674653

[B5] Ashina M. Doležil D. Bonner J. H. Zhou L. Klatt J. Picard H. (2021a). A phase 2, randomized, double-blind, placebo-controlled trial of AMG 301, a pituitary adenylate cyclase-activating polypeptide PAC1 receptor monoclonal antibody for migraine prevention. Cephalalgia. 41 (1), 33–44. 10.1177/0333102420970889 33231489 PMC7786389

[B6] Ashina M. Hansen J. á Dunga B. Olesen J. (2017). Human models of migraine — Short-term pain for long-term gain. Nat. Rev. Neurol. 13, 713–724. 10.1038/nrneurol.2017.137 28984313

[B7] Ashina M. Terwindt G. M. Al-Karagholi M. A. de Boer I. Lee M. J. Hay D. L. (2021b). Migraine: Disease characterisation, biomarkers, and precision medicine. Lancet 17397 (10283), 1496–1504. 10.1016/S0140-6736(20)32162-0 33773610

[B8] Bailey D. M. Taudorf S. Berg R. M. Jensen L. T. Lundby C. Evans K. A. (2009). Transcerebral exchange kinetics of nitrite and calcitonin gene-related peptide in acute mountain sickness: Evidence against trigeminovascular activation? Stroke 40, 2205–2208. 10.1161/STROKEAHA.108.543959 19359638

[B9] Bärtsch P. Bailey D. M. Berger M. M. Knauth M. Baumgartner R. W. (2004). Acute mountain sickness: Controversies and advances. High. Alt. Med. Biol. 5, 110–124. 10.1089/1527029041352108 15265333

[B10] Bärtsch P. Maggi S. Kleger G. R. Ballmer P. E. Baumgartner R. W. (1994). Sumatriptan for high-altitude headache. Lancet 344, 1445. 10.1016/s0140-6736(94)90617-3 7968111

[B11] Bärtsch P. Swenson E. R. (2013). Clinical practice: Acute high-altitude illnesses. N. Engl. J. Med. 368, 2294–2302. 10.1056/nejmcp1214870 23758234

[B12] Benemei S. De Cesaris F. Fusi C. Rossi E. Lupi C. Geppetti P. (2013). TRPA1 and other TRP channels in migraine. J. Headache Pain 13, 1471. 10.1186/1129-2377-14-71 PMC3844362 23941062

[B13] Borkum J. M. (2021). Brain energy deficit as a source of oxidative stress in migraine: A molecular basis for migraine susceptibility. Neurochem. Res. 46 (8), 1913–1932. 10.1007/s11064-021-03335-9 33939061

[B14] Borkum J. M. (2016). Migraine triggers and oxidative stress: A narrative review and synthesis. Headache 56, 12–35. 10.1111/head.12725 26639834

[B15] Britze J. Arngrim N. Schytz H. W. Ashina M. (2016). Hypoxic mechanisms in primary headaches. Cephalalgia 37, 372–384. 10.1177/0333102416647037 27146279

[B16] Burtscher M. Likar R. Nachbauer W. Schaffert W. Philadelphy M. (1995). Ibuprofen versus sumatriptan for high-altitude headache. Lancet 346, 254–255. 10.1016/s0140-6736(95)91303-3 7616830

[B17] Burtscher M. Mairer K. Wille M. Broessner G. (2010). Risk factors for high-altitude headache in mountaineers. Cephalalgia 31, 706–711. 10.1177/0333102410394678 21220379

[B18] Burtscher M. Wille M. Menz V. Faulhaber M. Gatterer H. (2014). Symptom progression in acute mountain sickness during a 12-hour exposure to normobaric hypoxia equivalent to 4500 M. High. Alt. Med. Biol. 15, 446–451. 10.1089/ham.2014.1039 25341048

[B19] Cernuda-Morollón E. Riesco N. Martínez-Camblor P. Serrano-Pertierra E. García-Cabo C. Pascual J. (2016). No change in interictal PACAP levels in peripheral blood in women with chronic migraine. Headache 56(9), 1448–1454. 10.1111/head.12949 27634731

[B20] Christiansen I. Thomsen L. L. Daugaard D. Ulrich V. Olesen J. (1999). Glyceryl trinitrate induces attacks of migraine without aura in sufferers of migraine with aura. Cephalalgia 19, 660–667. 10.1046/j.1468-2982.1999.019007660.x 10524660

[B21] Cohen A. S. Burns B. Goadsby P. J. (2009). High-flow oxygen for treatment of cluster headache: A randomized trial. JAMA 302, 2451–2457. 10.1001/jama.2009.1855 19996400

[B22] DaSilva A. F. Granziera C. Touch D. S. Snyder J. Vincent M. Hadjikhani N. (2007). Interictal alterations of the trigeminal somatosensory pathway and periaqueductal gray matter in migraine. Neuroreport 5, 301–305. 10.1097/WNR.0b013e32801776bb PMC3745625 17435592

[B23] Demartini C. Greco R. Zanaboni A. M. Sances G. De Icco R. Borsook D. (2019). Nitroglycerin as a comparative experimental model of migraine pain: From animal to human and back. Prog. Neurobiol. 177, 15–32. 10.1016/j.pneurobio.2019.02.002 30771365

[B24] Edvinsson L. (2021). CGRP and migraine: From bench to bedside. Rev. Neurol. 177 (7), 785–790. 10.1016/j.neurol.2021.06.003 34275653

[B25] Edvinsson L. Haanes K. A. Warfvinge K. (2019). Does inflammation have a role in migraine? Nat. Rev. Neurol. 15, 483–490. 10.1038/s41582-019-0216-y 31263254

[B26] Frank F. Faulhaber M. Messlinger K. Accinelli C. Peball M. Schiefecker A. (2020). Migraine and aura triggered by normobaric hypoxia. Cephalalgia 40 (14), 1561–1573. 10.1177/0333102420949202 32791920 PMC7838593

[B27] Frank F. Kaltseis K. Messlinger K. Broessner G. (2022). Short Report of longitudinal CGRP-measurements in migraineurs during a hypoxic challenge. Front. Neurol. In Press. 10.3389/fneur.2022.92574 PMC9367467 35968307

[B28] Goadsby P. J. Edvinsson L. Ekman R. (1988). Release of vasoactive peptides in the extracerebral circulation of humans and the cat during activation of the trigeminovascular system. Ann. Neurol. 23, 193–196. 10.1002/ana.410230214 2454066

[B29] Goadsby P. J. Holland P. R. Martins-Oliveira M. Hoffmann J. Schankin C. Akerman S. (2017). Pathophysiology of migraine: A disorder of sensory processing. Physiol. Rev. 97, 553–622. 10.1152/physrev.00034.2015 28179394 PMC5539409

[B30] Hansen J. M. Thomsen L. L. Marconi R. Casari G. Olesen J. Ashina M. (2008). Familial hemiplegic migraine type 2 does not share hypersensitivity to nitric oxide with common types of migraine. Cephalalgia 28, 367–375. 10.1111/j.1468-2982.2008.01542.x 18294248

[B31] Harris A. D. Roberton V. H. Huckle D. L. Saxena N. Evans C. J. Murphy K. (2013). Temporal dynamics of lactate concentration in the human brain during acute inspiratory hypoxia. J. Magn. Reson. Imaging 37, 739–745. 10.1002/jmri.23815 23197421 PMC3578150

[B32] Hasbak P. Lundby C. Olsen N. V. Schifter S. Kanstrup I. (2002). Calcitonin gene-related peptide and adrenomedullin release in humans: Effects of exercise and hypoxia. Regul. Pept. 108 (2-3), 89–95. 10.1016/s0167-0115(02)00129-5 12220731

[B33] Headache Classification Committee of the International Headache Society (IHS) (2018). Headache classification committee of the international headache society (IHS) the international classification of headache disorders, 3rd edition. Cephalalgia 38, 1–211. 10.1177/0333102417738202 29368949

[B34] Hughes R. Dake J. (1888). A cyclopedia of drug pathogenesy. New York: Boerike and Tafel.

[B35] Iversen H. K. (1995). Experimental headache in humans. Cephalalgia. 15, 281–287. 10.1046/j.1468-2982.1995.1504281.x 7585924

[B36] Jafarian S. Gorouhi F. Salimi S. Lotfi J. (2007). Sumatriptan for prevention of acute mountain sickness: Randomized clinical trial. Ann. Neurol. 62 (3), 273–277. 10.1002/ana.21162 17557349

[B37] Jaillard S. Mazetti P. Kala E. (1997). Prevalence of migraine and headache in a high-altitude town of Peru: A population-based study. Headache 37, 95–101. 10.1046/j.1526-4610.1997.3702095.x 9074294

[B38] Kc P. Dick T. E. (2010). Modulation of cardiorespiratory function mediated by the paraventricular nucleus. Respir. Physiol. Neurobiol. 174, 55–64. 10.1016/j.resp.2010.08.001 20708107 PMC2967641

[B39] Keith I. M. Ekman R. (1992). Dynamic aspects of regulatory lung peptides in chronic hypoxic pulmonary hypertension. Exp. Lung Res. 18 (2), 205–224. 10.3109/01902149209031681 1572330

[B40] Keith I. M. Tjen-A-Looi S. Kraiczi H. Ekman R. (2000). Three-week neonatal hypoxia reduces blood CGRP and causes persistent pulmonary hypertension in rats. Am. J. Physiol. Heart Circ. Physiol. 279 (4), H1571–H1578. 10.1152/ajpheart.2000.279.4.H1571 11009443

[B41] Konrad F. Moritz A. Moritz M. Keunecke J. G. Tischler F. Prottengeier J. (2022)., THe epidemiology of airplane headache: A cross-sectional study on point prevalence and characteristics in 50,000 travelers, Cephalalgia, 3331024221092408. Epub ahead of print. 10.1177/03331024221092408 PMC9442279 35414200

[B42] Kraenzlin M. E. Ch’ng J. L. C. Mulderry P. K. Ghatei M. A. Bloom S. R. (1985). Infusion of a novel peptide, calcitonin gene-related peptide (CGRP) in man. Pharmacokinetics and effects on gastric acid secretion and on gastrointestinal hormones. Regul. Pept. 10, 189–197. 10.1016/0167-0115(85)90013-8 3922013

[B43] Messlinger K. (2018). The big CGRP flood - sources, sinks and signalling sites in the trigeminovascular system. J. Headache Pain 19, 22. 10.1186/s10194-018-0848-0 29532195 PMC5847494

[B44] Moskowitz M. A. (1993). Neurogenic inflammation in the pathophysiology and treatmentof migraine. Neurology 43, 16 8389008

[B45] NosedaR.BursteinR. (2013). Migraine pathophysiology: Anatomy of the trigeminovascular pathway and associated neurological symptoms, cortical spreading depression, sensitization, and modulation of pain. Pain 154, 44–53. 10.1016/j.pain.2013.07.021 23891892

[B46] Ray B. S. Wolff H. G. (1940). Experimental studies on headache: Pain-sensitive structures of the head and their significance in headache. Arch. Surg. 41 (4), 813–856. 10.1001/archsurg.1940.01210040002001

[B47] Reuter U. Bolay H. Jansen-Olesen I. Chiarugi A. Sanchez del Rio M. Letourneau R. (2001). Delayed inflammation in rat meninges: Implications for migraine pathophysiology. Brain 124, 2490–2502. 10.1093/brain/124.12.2490 11701602

[B48] Rosenfeld M. G. Mermod J. J. Amara S. G. Swanson L. W. Sawchenko P. E. Rivier J. (1983). Production of a novel neuropeptide encoded by the calcitonin gene via tissue-specific RNA processing. Nature 304, 129–135. 10.1038/304129a0 6346105

[B49] Sances G. Tassorelli C. Pucci E. Ghiotto N. Sandrini G. Nappi G. (2004). Reliability of the nitroglycerin provocative test in the diagnosis of neurovascular headaches. Cephalalgia 24 (2), 110–119. 10.1111/j.1468-2982.2004.00639.x 14728706

[B50] Schoonman G. Sándor P. Agosti R. Siccoli M. Bärtsch P. Ferrari M. (2006). Normobaric hypoxia and nitroglycerin as trigger factors for migraine. Cephalalgia 26 (7), 816–819. 10.1111/j.1468-2982.2006.01112.x 16776696

[B51] Schulte L. H. May A. (2016). The migraine generator revisited: Continuous scanning of the migraine cycle over 30 days and three spontaneous attacks. Brain 139, 1987–1993. 10.1093/brain/aww097 27190019

[B52] Schytz H. W. Birk S. Wienecke T. Kruuse C. Olesen J. Ashina M. (2009). PACAP38 induces migraine-like attacks in patients with migraine without aura. Brain 132, 16–25. 10.1093/brain/awn307 19052139

[B53] Sicuteri F. Del Bene E. Poggioni M. Bonazzi A. (1987). Unmasking latent dysnociception in healthy subjects. Headache 27 (4), 180–185. 10.1111/j.1526-4610.1987.hed2704180.x 3110103

[B54] Somjen G. G. Aitken P. G. Czéh G. L. Herreras O. Jing J. Young J. N. (1992). Mechanism of spreading depression: A review of recent findings and a hypothesis. Can. J. Physiol. Pharmacol. 70, 248–254. 10.1139/y92-268 1295674

[B55] Takano T. Tian G. F. Peng W. Lou N. Lovatt D. Hansen A. J. (2007). Cortical spreading depression causes and coincides with tissue hypoxia. Nat. Neurosci. 10, 754–762. 10.1038/nn1902 17468748

[B56] Thomsen L. L. Olesen J. (1997). A pivotal role of nitric oxide in migraine pain. Ann. N. Y. Acad. Sci. 835, 363–372. 10.1111/j.1749-6632.1997.tb48642.x 9616786

[B57] Tuka B. Helyes Z. Markovics A. Bagoly T. Szolcsányi J. Szabó N. (2013). Alterations in PACAP-38-like immunoreactivity in the plasma during ictal and interictal periods of migraine patients. Cephalalgia 33 (13), 1085–1095. 10.1177/0333102413483931 23598374

[B58] van Dongen R. M. Zielman R. Noga M. Dekkers O. M. Hankemeier T. van den Maagdenberg A. M. (2017). Migraine biomarkers in cerebrospinal fluid: A systematic review and meta-analysis. Cephalalgia 37 (1), 49–63. 10.1177/0333102415625614 26888294

[B59] Waschek J. A. Baca S. M. Akerman S. (2018). PACAP and migraine headache: Immunomodulation of neural circuits in autonomic ganglia and brain parenchyma. J. Headache Pain 19 (1), 23. 10.1186/s10194-018-0850-6 29536279 PMC5849772

[B60] Zagami A. S. Edvinsson L. Goadsby P. J. (2014). Pituitary adenylate cyclase activating polypeptide and migraine. Ann. Clin. Transl. Neurol. 1 (12), 1036–1040. 10.1002/acn3.113 25574477 PMC4284128

